# Live animals for preclinical medical student surgical training

**Published:** 2016-12-15

**Authors:** Stephanie C. DeMasi, Eriko Katsuta, Kazuake Takabe

**Affiliations:** 1 Division of Surgical Oncology, Department of Surgery, Virginia Commonwealth University School of Medicine and Massey Cancer Center, Richmond, VA, USA; 2 Breast Surgery, Department of Surgical Oncology, Roswell Park Cancer Institute, Buffalo, NY, USA; 3 Department of Surgery, University at Buffalo, The State University of New York School of Medicine and Biomedical Sciences, Buffalo, NY, USA

**Keywords:** Animal models, Education, Mice, Mastectomy, Surgical skills, Training

## Abstract

**Aims:**

The use of live animals for surgical training is a well-known, deliberated topic. However, medical students who use live animals rate the experience high not only in improving their surgical techniques, but also positively influencing their confidence levels in the operating room later in their careers. Therefore, we hypothesized that the use of live animal models is a unique and influential component of preclinical medical education.

**Materials and Methods:**

Medical student performed the following surgical procedures using mice; surgical orthotopic implantation of cancer cells into fat pad and subsequently a radical mastectomy. The improvement of skill was then analyzed.

**Results:**

All cancer cell inoculations were performed successfully. Improvement of surgical skills during the radical mastectomy procedure was documented in all parameters. All wounds healed without breakdown or dehiscence. The appropriate interval between interrupted sutures was ascertained after fifth wound closure. The speed of interrupted sutures was doubled by last wound closure. The time required to complete a radical mastectomy decreased by almost half. A single animal died immediately following the operation due to inappropriate anesthesia, which was attributed to the lack of understanding of the overall operative management.

**Conclusion:**

Surgical training using live animals for preclinical medical students provides a unique learning experience, not only in improving surgical skills but also and arguably most importantly, to introduce the student to the complexities of the perioperative environment in a way that most closely resembles the stress and responsibility that the operating room demands.

## INTRODUCTION

Medical students entering clinical rotations following two years of didactic lecturing are enthusiastic to integrate the knowledge they have gained, finally, in the clinical setting. However, many feel that medical school curricula are not meeting these amplified expectations and desires to learn these surgical skills, which students feel is an imperative component of their medical education [1, 2]. Traditionally, exposure to surgery in the preclinical years of medical school has been remarkably limited. Yet research has shown that relying solely on clerkships in third and fourth year of medical school to teach procedural skill is inadequately preparing students for their future practice [3]. Recently, it has also shown to result in a negative connotation towards of the field of surgery with consequent low application rates to categorical surgical residency programs [4]. Restriction is not only limited in time but also in its location, which conventionally has been in the operating room, which presents a unique challenge [1]. The atmosphere of the operating room invites barriers that can contribute to medical student’s anxiety, low confidence, and poor self-assessment early in their clinical training [1, 5]. It has been described that the ever growing pressures and difficult working conditions in complex sub-specialized surgical fields causes dissatisfaction among young residents, let alone the medical students [6]. In addition, surgical training of preclinical medical students presents a binding concern to the safety of patients being operated on by students with limited, if any, surgical training [7].

To overcome these challenges, medical schools have implemented procedural workshops in a simulated setting to provide instruction outside of the operating room. Use of live animal models to train medical students has long been a topic of debate [6, 8]. The advantages of the use of live animals are many. They are appreciated as the best means to simulate patients in terms of preparing medical students for the anxiety and extensive perioperative demands required in the operating room, as a life is at stake even if it is an animal’s [1, 6]. Some believe that animal models are beneficial for training students, as it affords an opportunity for innovation and critical thinking because the animals have very different anatomy [6]. Counter arguments on the use of live animals during student training include ethical concerns, extensive cost to obtain and house the animals, and absence of faculty to provide instruction [6]. Presently, only 1 in 5 of medical school procedural workshops use live animals [6]. However, recent studies have shown that medical students who had the opportunity to use live animals in their training rated the experience exceedingly high; not only in improving their surgical techniques, but also positively influencing their attitudes and confidence levels in the operating room later in their training, and potentially increasing their interest in surgery as a career [1, 6]. A study by Daly et al. found over 90% of students reported that they felt training in an animal laboratory was an imperative part of their education and that it should be included in the future curriculum [1, 6]. In agreement, Liddell et al. found that a single session of teaching procedural skills in the early stages of medical school may improve confidence and long-term effectiveness in basic skills [3].

With the features of current medical student education and surgical training in context, the aim of our study was to examine the effects of surgical training using mice for medical students before any clinical exposure.

## MATERIALS AND METHODS

### Participant

All surgical procedures were performed by a second year medical student at Virginia Commonwealth University, whom had passed all preclinical examinations but had yet to participate in any clinical rotations, including all surgical sub-internships.

### Animal Study

All animal studies were conducted in the Animal Research Core Facility at Virginia Commonwealth University School of Medicine in accordance with the institutional guidelines. All surgical procedures were approved by the Virginia Commonwealth University Institutional Animal Care and Use Committee (IACUC), accredited by the Association for Assessment and Accreditation of Laboratory Animal Care, and all protocols were followed. Female C57BL/6 and BALB/c mice, 20 to 40 weeks of age, weighing between approximately 15 g and 25 g were obtained from Jackson Lab. General anesthesia was induced and maintained with inhaled isoflurane. Continuous monitoring of the respiratory rate was observed throughout the procedure. Animals were closely monitored in the perioperative period to observe for signs of distress. Standard surgical instruments including scissors, forceps, hemostat, scalpel, and 5-0 silk ties, were supplied. All procedures were performed in an aseptic environment. A didactic session of the basic surgical technique was demonstrated and supervised by a board certified attending surgeon. The student observed one procedure demonstrating each skill prior to the start of their assessment.

### Tumor cell implantation model

The first procedure was designed to introduce basic surgical skill by way of orthotopic implantation of 4T1-luc2 murine mammary adenocarcinoma cells (1×10^4^ cells suspended in 20 µl Matrigel) into bilateral #2 (chest) mammary fat pad under direct vision as we previously reported [9, 10]. Under inhaled anesthesia, a 5-mm midline incision was made medial to the #2 nipple, and a sterile cotton swab was used to expose the mammary gland. A 27-gauge needle was used to implant the 4T1-luc2 cells directly into the gland.

### Radical Mastectomy Model

A second more technically complex procedure was the radical mastectomy, defined as the surgical removal of the tumor with surrounding skin and pectoralis major muscle. Tumors were generated into bilateral #2 fat pads 14 days prior this operation. Bilateral mammary fat pads were palpated from the surface of the anterior chest wall. The fur was then clipped, and the animal was anesthetized with inhaled isoflurane. While induced the animal was prepped and draped in an aseptic manner. A 5-mm midline incision was made medial to the #2 nipple and a sterile cotton swab used to raise the skin flap by pressing the cotton swab against the anterior chest wall. The vessels in the area were cut, with special care to avoid the axillary vessels which could result in massive bleeding and consequent animal death. The mammary gland, adjacent skin, and underlying pectoralis major muscle were separated from adherent tissues and were excised. Then skin was closed with interrupted stitches using the basic technique learned from the first laboratory exercise.

### Evaluation

Tumor formation rate was evaluated in tumor implantation model. Length and width of wound, time to complete procedure from first incision to final suture, number of sutures per cm wound, and weight of tissue removed were evaluated in mastectomy model. Perioperative management was assessed preoperatively and the cost of the study was analyzed.

## RESULTS

### Murine orthotopic implantation under direct vision

Basic surgical technique was introduced by way of a murine breast cancer model with orthotopic implantation of murine cancer cells under direction vision. In cancer cell injection technique, when wound was closed, inoculation site was compressed and a part of Matrigel spilled out from wound in first case. However, all inoculation (5 out of 5) formed tumor successfully. There were no perioperative or postoperative complications observed. After a total of five operations, which included successful closure of five wounds and approximately 30 suture ties, the student advanced to the next procedure.

### Radical Mastectomy Model

**Figure 1:**
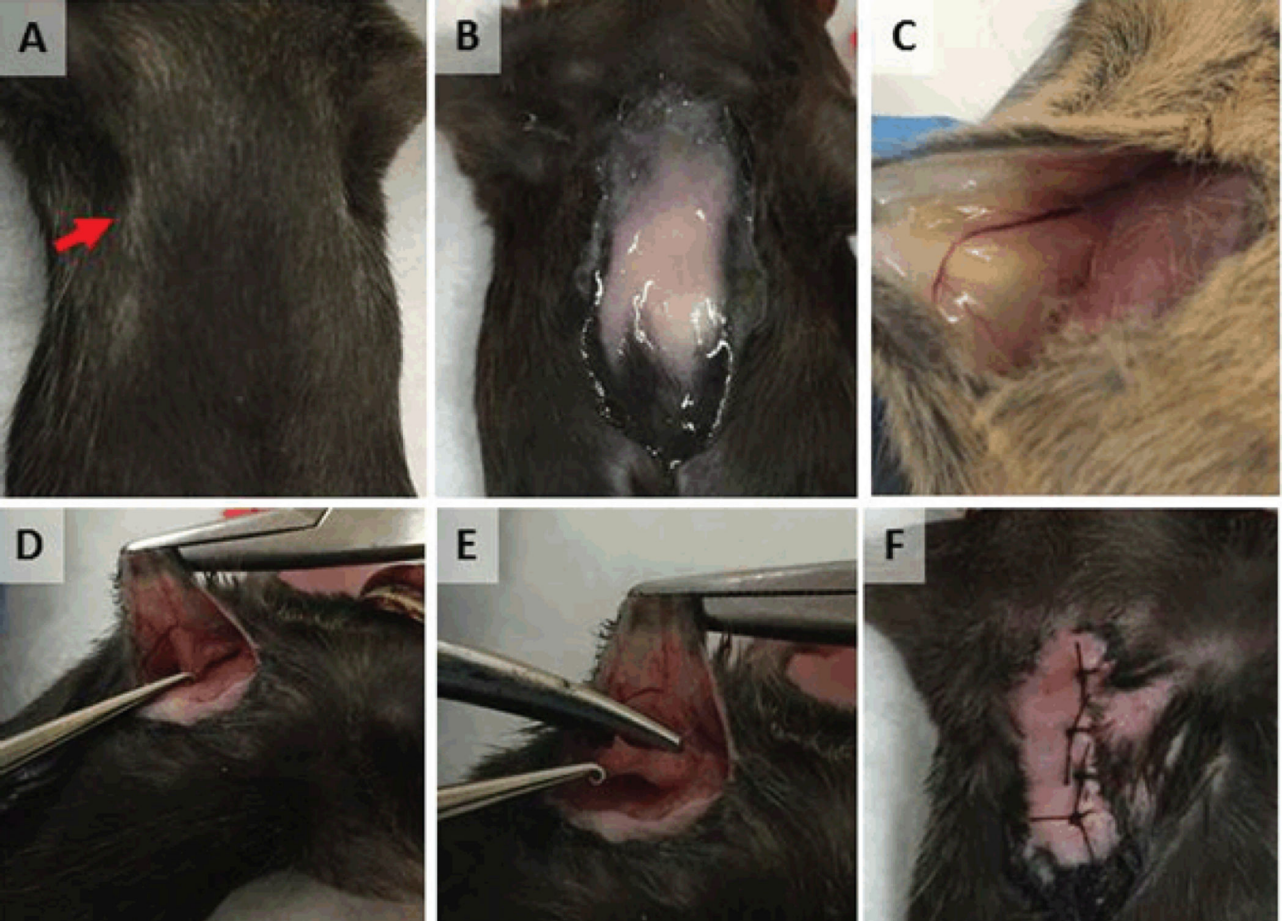
Radical mastectomy of murine breast cancer. (A) The #2 (chest) nipples were identified on the anterior chest wall. (B) Fur was clipped, animal was anesthetized with inhaled isoflurane, and was prepped and draped in an aseptic manner. (C) 5mm midline incision was made medial to the #2 nipple and a sterile cotton swab used to develop skin flap. (D) Mammary gland, adjacent skin, and underlying pectoralis major muscle was separated from adherent tissues and (E) were excised. (F) Skin was closed with interrupted stitches.

**Figure 2:**
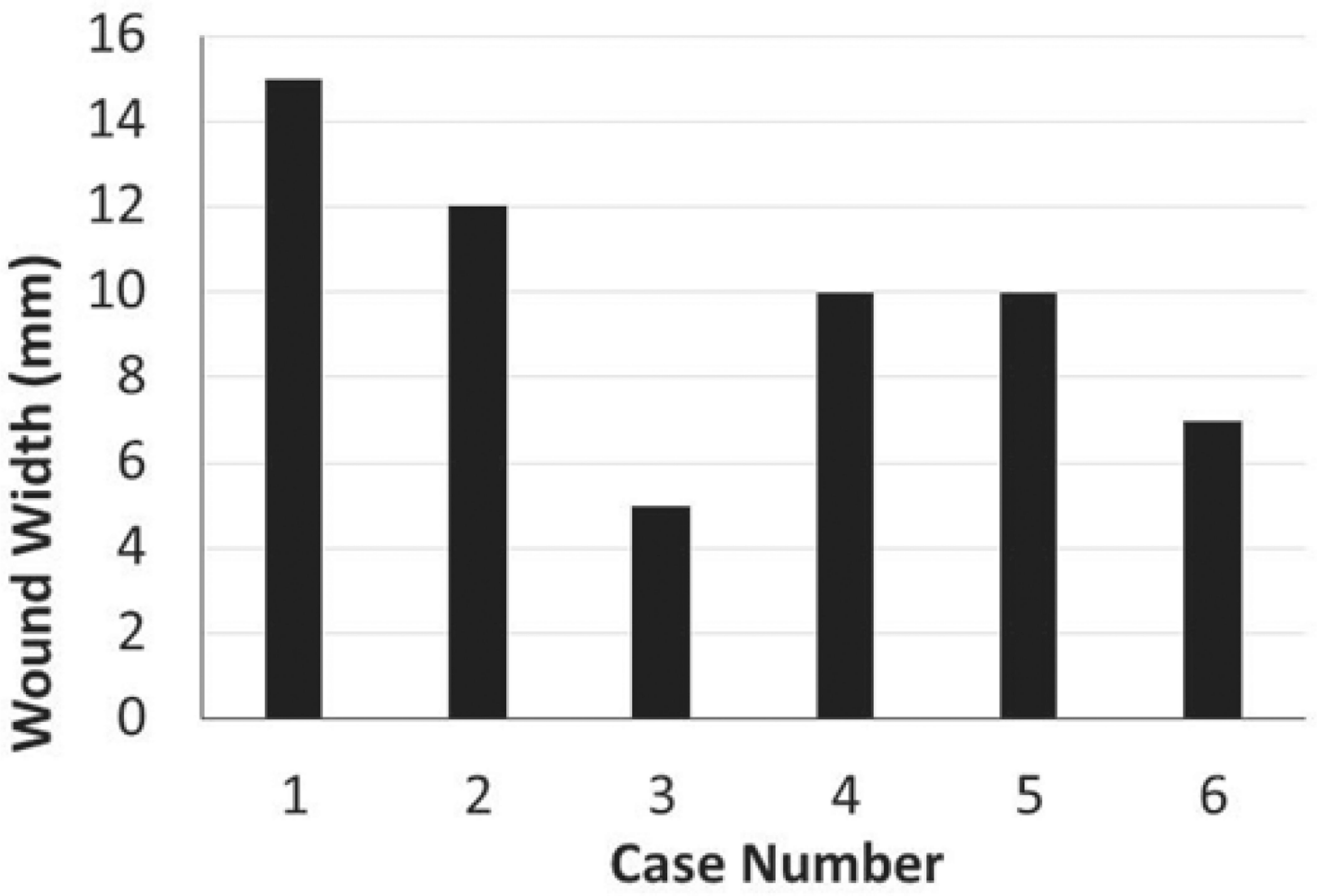
Wound closure was successful regardless of the width. Widths of the wounds varied depending on the case and amount of tissue resection needed, and ranged from 15 mm noted on the first wound closure experience, to 5 mm. All the wounds were successfully closed and healed regardless of the widths.

**Figure 3:**
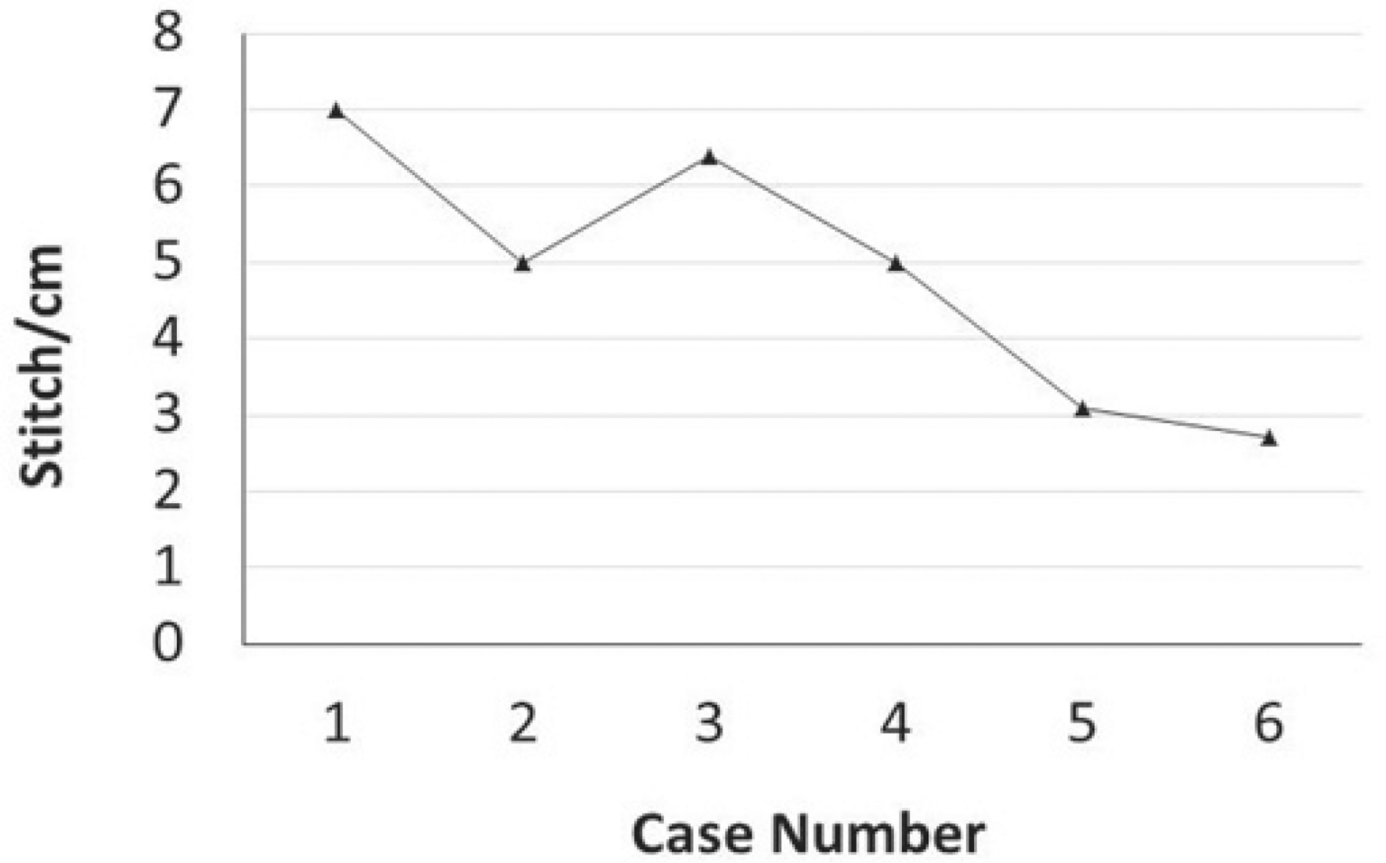
The appropriate stitch interval was ascertained after fifth wound closure. It was found during the initial cases that the interval between sutures was inappropriately tight and close together, which improved in latter cases.

Radical mastectomies for bilateral #2 fat pads were observed and subsequently performed (Figure 1). Relatively complex surgical model, radical mastectomy, allowed for an analysis of the previously learned basic surgical skills; closure of widely open wounds, number of interrupted sutures necessary to close the wound, and time it took to complete each procedure. Significant improvement of surgical skills was observed in all parameters recorded. It was found that wound closure was successful in all widths attempted, despite the fact that some wound widths were up to 15 mm (Figure 2). This was a rather unexpected finding for an inexperienced student, as one would anticipate either inability to close wounds of large dimension, or wound break down or dehiscence due to loosened ties. On the other hand, what was found during the initial cases was that the interval between sutures was inadequately spaced between one another, and the sutures were closed inappropriately tight, which may have helped in avoiding the wound break down with a cost in extending the procedure time and possible ischemia of the wound edges. The appropriate stitch interval was ascertained after the fifth wound closure (Figure 3).

**Figure 4:**
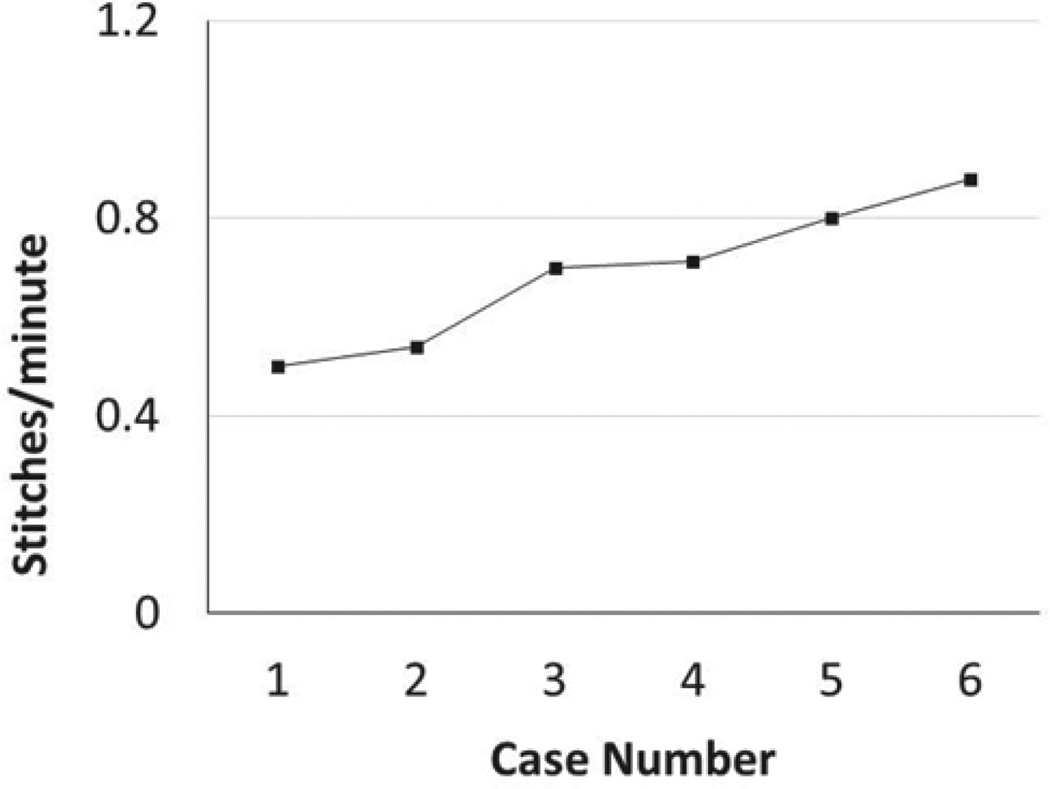
The number of stitches per minute increased. The speed of suturing was roughly 1 interrupted stitch every two minutes in the first wound, however, improvement was observed after each would closure, and the time to complete a successful interrupted stitch decreased by half after the sixth wound closure.

The speed of suturing during the initial procedure was roughly 1 interrupted suture per 2 minutes. However, improvement was observed after each would closure, and the time to complete a successful interrupted suture decreased by half after only six wound closure experiences (Figure 4).

**Figure 5:**
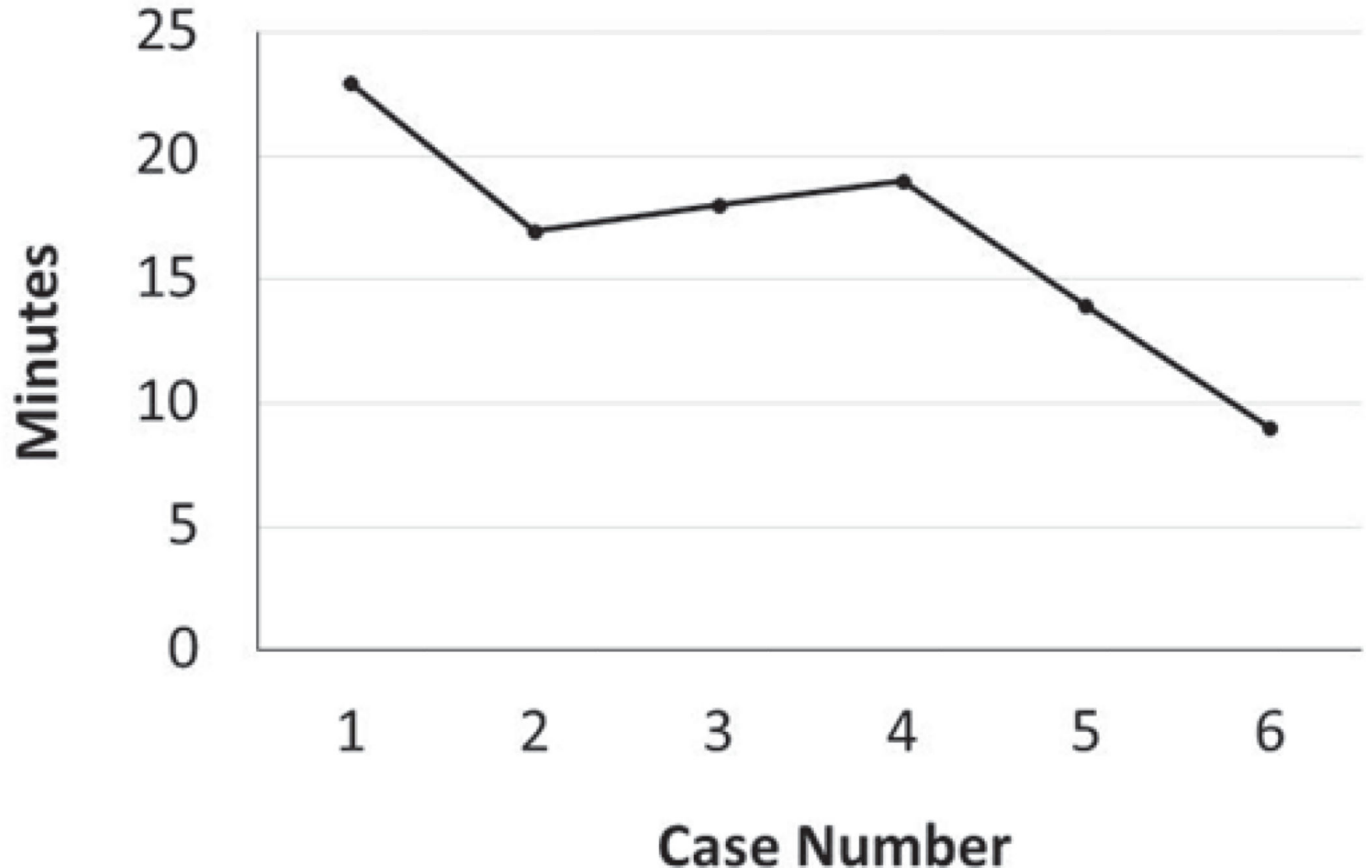
The time required to perform a complete radical mastectomy decreased by greater than half. Initially, it took almost 23 minutes to complete the procedure. As skill of suturing improving, combined with comfort level and confidence, improvement in time was documented with each case. The time to complete the procedure decreased almost half by the last case. The final procedure took only nine minutes to complete.

The time to complete a radical mastectomy initially took an ample 23 minutes. As skill of suturing improved, combined with increasing comfort level and confidence with practice, improvement in time was documented with each case. It was found that the time to complete a radical mastectomy decreased by almost half by the 6th case, the final procedure took only nine minutes to complete in its entirety (Figure 5). This was a substantial decrease in time from the first operation.

### Perioperative Management

**Figure 6:**
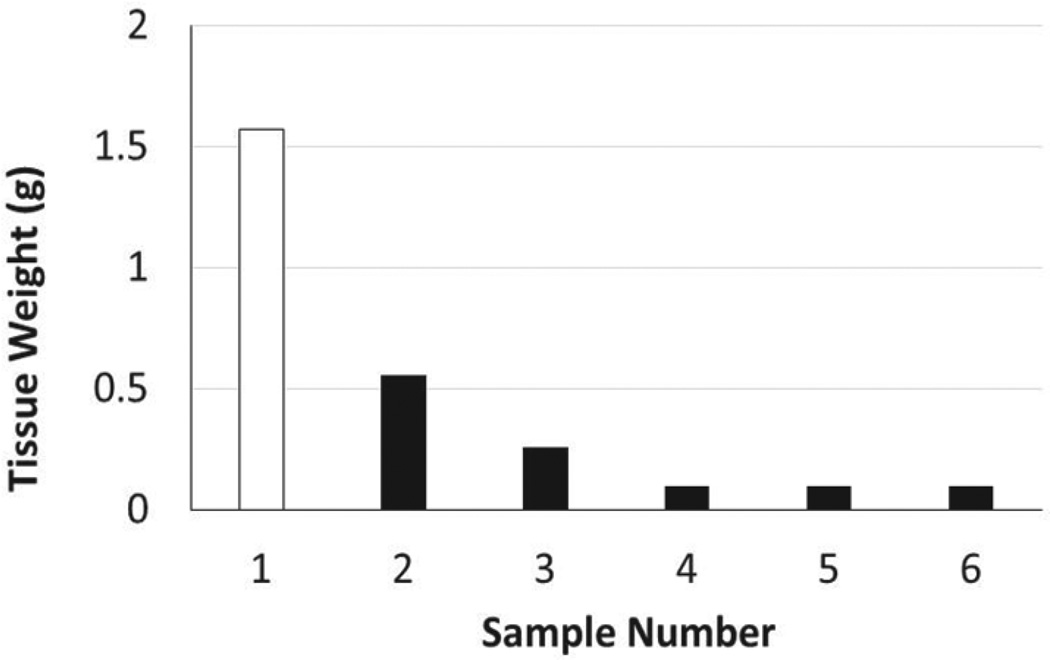
Recognition of the importance of life-support and perioperative management during an operation. A single animal died immediately after the operation (indicated by white), which was determined to be a result of inappropriate intraoperative monitoring and depth of anesthesia. Extra time was needed to complete this procedure, as this specific resection was of a very large tumor, at least 3-fold higher in weight than all other cases recorded. The death of the animal was attributed to the lack of understanding of the perioperative management.

Radical mastectomy certainly required complex surgical technique for a preclinical medical student, in terms of the microsurgical skills needed to remove the adherent skin, controlling the bleeding following severed blood vessels, as well as utilizing the proper suture tying technique. Notably, a single animal (Case 1) died immediately after the operation, which was retrospectively determined to be a result of inappropriate anesthesia management. Extra time was needed to complete this procedure, in particular, as compared to other cases. The tumor was very large (Figure 6) and the speed of the preoperative preparation was inappropriately slow since this was the first procedure, which included induction of anesthesia, hair removal, and sterile draping. This was in addition to the underdeveloped surgical skill that supplemented the lengthy total operation time that resulted in poor respiratory monitoring and subsequent lethal depth of anesthesia. This was contrary to the anticipated cause of perioperative death, which was thought to be more likely due to failure to control bleeding, pneumothorax, or wound dehiscence, when performed by a student with inexperienced surgical skills. However, it was in fact the lack of understanding of the overall operative management and importance of life-support that actually resulted in the death of the animal.

### Financial Analysis

A cost analysis of all materials used in the present study revealed financial obligation of roughly $50.00 per mouse, with a total financial obligation for one procedure per student roughly $157. The inclusion for the entire 2nd year medical school class of about 200 students, the cost is approximately $12,143 (Table 1).

## DISCUSSION

There is an undisputed requisite for basic surgical skills training in preclinical medical education. In part due to the increasing dissatisfaction of young residents coupled with the growing complexity of surgical training, as well as the imperious concern for patient safety. The lack of proper training can exacerbate the challenges presented in the high pressure environment of the operating room, which contributes to the stress, anxiety, and low self-assessment of medical students in early clinical rotations [1, 3, 5, 6, 11]. Medical students have steadily shown either major deficits or wide variability in basic surgical skills [6, 12, 13]. The recent shortfalls of traditional training have been attributed to the financial burden, as well duty hour limitations resulting in lack of adequate time to instruct students, as teaching in the operating room can double procedure time [6, 14, 15].

Medical training programs have implemented a variety of programs in an attempt to overcome these challenges. These include non-biological models, ex vivo animal tissue models, and in vivo animals in a simulated setting. Inorganic models include human simulation centers and non-biological tissue models. Some institutions offer an elective course on surgical training skills at the human simulation center to learn suturing, knot tying, laparoscopic technique, management of perioperative complications, and teamwork in the operating room. Human simulation centers have had some success in terms of objective improvement of skill, however the average start-up cost is approximately $450,000 [16]. Furthermore, annual maintenance expenditures can range from $12,000 to $300,000 [16]. A non-biological model taught flap and Z-plasty techniques using a foam rubber model [17]. However, non-biological models of teaching are encumbered with the trivial question; how closely do they truly simulate human tissue and the actual clinical environment? Though these models are cheap and easy to provide, studies have shown that non-biological models do not simulate human tissue as closely nor do they accurately represent the clinical situation when matched with animal tissue models [7, 15]. However, if compared to didactic lessons only or as a stepwise progression to animal model training it has shown that it can provide some benefit [17, 18].

Didactic lessons combined with an ex vivo pig model to teach neck dissection, radial forearm free flap, microvascular anastomosis, split-thickness skin graft, Z-plasty, and local flaps resulted improvement in surgical techniques across the board following their teaching method [7]. An ex vivo study using pig skin flaps that included stations to teach basic suturing, IV access, wound debridement, and closure of lacerations, as well as an in vivo pig model found improvements in the ex vivo objective post-assessment analysis of basic surgical skills, however concluded that in vivo models would be the ideal high-fidelity simulation environment [19]. Notably ex vivo models are a considerably cost-effective method of training, however deep skin techniques are not possible to emanate from this design [7, 19]. Large, live animal models have proven to better simulate human tissue and operating room environment as well as provide for more complex instruction and surgical technique acquisition [7]. Using live swine models that included instruction of laparotomy, small bowel resection, splenectomy, cholecystectomy, among others, found significant improvement across all post-test scores as well as positive feedback from the students whom participated [1].

There is an obligatory notion that animal models better simulate the stress and responsibility that the operating room demands. Specifically, for the past three decades it has been well recognized that poor airway management poses a serious threat to patient safety [20]. Therefore, the animal model provides a huge advantage and an additional learning curve that non-biological models, or even ex vivo models, do not offer. This was exemplified in our study as an animal’s life was compromised due to that very fact and the student was able to experience the various complex components that need to be carefully managed during a surgical procedure, where a life is at stake, even if it is an animal’s. This unique learning experience may in fact be more beneficial in one’s training than all other objective measurements of skill acquisition combined. The limitations of large, live animal models include ethical duties, financial obligation, and absence of faculty [6]. Thus, our study aims to compromise the cost of large animal models versus non-biological models, by using a smaller murine animal model; proposing the benefits are largely equivalent. The average cost per pig alone is approximately $608, whereas mice cost roughly from $80 to $90 [1, 21]. The success of our simulated skills lab was evidenced by the significant improvement in all post-test surgical skills. The outcomes also detailed adjustment to the perioperative environment, an imperative aspect of surgical training thus proving that this model depicts a similar environment as the operating room. All surgical techniques were easily performed with practice, by the student. Though the ethical concern of the use of animal tissue still remains, one must weigh the potential compromise of patient safety when operated on by inexperienced students and/or young physicians, which also presents an ethical dilemma in it of itself. Notwithstanding, limitations are notable in this study. The first being the study was limited to a single medical student during a summer fellowship program and thus a large number of students could not participate as needed. Second, a control group comparing different surgical technique models was absent. Future investigations should include both a larger number of students to participate, as well as a control group for comparison of findings.

## CONCLUSION

The live animal model, even a small, inexpensive murine model, to train preclinical medical students provides a unique learning experience, not only to improve surgical skills, but also, and arguably most importantly, to teach the student to appreciate and understand the complexities of the perioperative environment, such as anesthesia management, that are critical to maintaining life.

## Figures and Tables

**Table 1:** Financial obligation for Murine laboratory

Variable	Cost
Animal Housing	$0.14 /mouse/day
Anesthesia (Isoflurane 250ml bottle)	$25.00
Surgical Materials	$104.00
Price per mouse	$10.44
Shipping per mouse	$40.00
Total for one procedure per student	$157.71
Entire 2nd Year Medical Student Class	$12143.00
